# Effect of remimazolam besylate versus midazolam on time to extubation in critically ill, mechanically ventilated patients: a randomized controlled trial

**DOI:** 10.3389/fmed.2025.1553495

**Published:** 2025-08-26

**Authors:** Renhuai Liu, Binxiao Su, Guifen Gan, Guangming Wang, Chengli Wang, Ning Xu, Guangcai Feng, Hao Guo, Qingxia Yuan, Aiguang Li, Wenping Zheng, Jiang Li, Yu Chen, Xijing Zhang

**Affiliations:** ^1^Department of Critical Care Medicine, Xijing Hospital, Fourth Military Medical University, Xi’an, Shaanxi, China; ^2^Department of Critical Care Medicine, Affiliated Hospital of Qinghai University, Xining, China; ^3^Department of Critical Care Medicine, Norinco General Hospital, Xi’an, Shaanxi, China; ^4^Department of Critical Care Medicine, 3201 Hospital, Hanzhong, Shaanxi, China; ^5^Department of Critical Care Medicine, The Second Affiliated Hospital of Yan’an University, The First Hospital of Yulin, Yulin, Shaanxi, China; ^6^Department of Intensive Care Medicine, Ankang Traditional Chinese Medicine Hospital, Ankang, Shaanxi, China; ^7^Department of Critical Care Medicine, Xianyang First People's Hospital, Xianyang, Shaanxi, China; ^8^Department of Intensive Care Unit, Xi'an International Medical Center Hospital, Xi’an, Shaanxi, China; ^9^Department of Critical Care Medicine, Xi'an Aerospace General Hospital, Xi’an, Shaanxi, China; ^10^Department of Neurosurgery, Hancheng People's Hospital, Hancheng, Shaanxi, China; ^11^Department of Critical Care Medicine, Ankang Central Hospital, Ankang, Shaanxi, China

**Keywords:** remimazolam besylate, midazolam, sequential sedation, intensive care, mechanical ventilation

## Abstract

**Background:**

Previous studies have indicated that the administration of short-acting sedatives prior to weaning from mechanical ventilation is linked to a more rapid recovery and extubation process, in addition to lowering intensive care unit (ICU) treatment expenses. The present study aimed to evaluate the efficacy and safety of the sequential administration of remimazolam besylate compared with midazolam before weaning from mechanical ventilation.

**Methods:**

This multicenter, randomized controlled trial was conducted across medical and surgical ICUs within a tertiary, academic medical center. The study population consisted of critically ill, mechanically ventilated adult patients. Candidates anticipated to be ready for ventilator weaning within 12 h underwent a Spontaneous Breathing Trial (SBT) safety screen. Only those who successfully passed this assessment were considered for inclusion in the final phase of the study and subsequent randomization. The patients were randomized into two groups: group M, in which the sedative regimen was transitioned to midazolam, and group R, which involved a switch to remimazolam. Sedative dosages were titrated to achieve a target Richmond Agitation-Sedation Scale (RASS) score between −3 and 0. The primary endpoint of this study was the time to extubation.

**Results:**

A total of 435 patients underwent screening, of whom 306 patients being randomized, and 272 patients ultimately included in the analysis, comprising 132 patients in group M and 140 patients in group R. The patients in group R maintained lighter levels of sedation compared to those in group M. The patients in group R showed significantly earlier recovery (*p* < 0.05) and extubation (*p* < 0.05) at the same RASS score prior to the cessation of sedatives. Higher prevalence of agitation was observed in group M as opposed to group R (20.45% versus 8.57%, *p* = 0.005). However, no significant difference in the incidence of delirium was noted between the groups.

**Conclusion:**

In critically ill, mechanically ventilated patients, the use of remimazolam besylate was associated with a shorter time to recovery and extubation prior to ventilator weaning, along with a lower incidence of agitation.

**Clinical trial registration:**

Identifier ChiCTR 2200065048, https://www.chictr.org.cn.

## Background

Mechanical ventilation (MV) is a critical, life-supporting intervention that is widely used in intensive care units (ICUs), with an estimated 20 million patients globally requiring this intervention annually (1). Sedation plays a crucial role in the management of mechanically ventilated patients, as it helps mitigate anxiety and agitation, provides amnesia, thereby enhancing overall patient comfort (2).

Propofol and midazolam are commonly administered as first-line sedatives to mechanically ventilated patients (3). However, these drugs exhibit distinct side-effect profiles and problems during prolonged sedation (4). Midazolam, a potent anxiolytic, hypnotic, and sedative agent, is associated with the unpredictable accumulation of its active metabolite and may potentially induce anterograde amnesia. Nevertheless, studies suggest that sedation with midazolam in mechanically ventilated patients is associated with a high risk of delayed recovery, prolonged mechanical ventilation, and delirium. Although clinical guidelines advocate for light sedation with non-benzodiazepine sedatives (5), midazolam remains widely used in clinical practice (2). Recent surveys have shown that a sequential sedation strategy, using various sedatives, yields superior clinical outcomes and fewer adverse events when compared to mono-sedative regimens in long-term sedation of critically ill, mechanically ventilated patients (6).

Remimazolam besylate is a novel, ultra-short-acting benzodiazepine that rapidly metabolizes through non-specific tissue esterases into an inactive carboxylic acid metabolite, offering a rapid and predictable onset and offset (7). Prolonged infusions or higher doses are unlikely to result in accumulation and an extended effect. It can also be safely administered in patients with compromised liver or renal function.

Previous studies have shown that the administration of short-acting sedatives before weaning from mechanical ventilation is associated with faster recovery and extubation, in addition to reduced ICU treatment costs (8). Building on this, it was hypothesized that the sequential use of midazolam and remimazolam besylate during the weaning process could improve clinical outcomes. The aim of this study was designed to evaluate the efficacy and safety of the sequential use of remimazolam besylate as compared with midazolam in patients undergoing weaning from mechanical ventilation.

## Methods

### Study design

This multi-center, prospective, randomized, controlled pilot study was approved by the Ethics Committee of Xijing Hospital (KY20222207-C-1). Written informed consent was obtained from all patients or their legal representatives. This study was registered before enrollment at clinicaltrials.gov (ChiCTR2200065048).

### Patients

**Figure 1:**
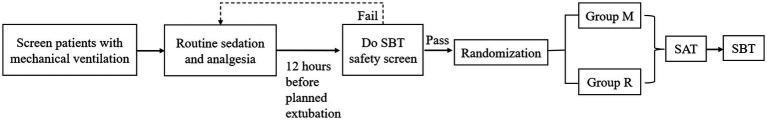
Study protocol. SAT, Spontaneous Awakening Trial; SBT, Spontaneous Breathing Trial.

Patient recruitment was conducted through a two-stage process, comprising an initial screening phase followed by a confirmatory phase (Figure 1). All patients admitted to the ICUs requiring intubation, mechanical ventilation, or those intubated during their ICU stay were followed. The inclusion criteria for patient selection were as follows: intubated patients aged between 18 and 80 years who were expected to require mechanical ventilation for 24 h or more following ICU admission. The exclusion criteria encompassed known or suspected allergy to remimazolam besylate or midazolam, suspected pregnancy, morbid obesity (body mass index ≥30 kg/m^2^), moribund state, history of alcoholism or current use of anxiolytic or hypnotic medications, chronic hepatic failure chronic renal failure, anticipated difficulty in weaning from ventilator (e.g., high spinal cord injury or myasthenia gravis), coma due to cranial trauma or neurosurgery or of unknown etiology, status epilepticus, enrollment in other studies within 3 months, and rejected to provide informed consent by patient or their authorized surrogates following ICU admission. The diagnostic criteria for liver function injury, chronic hepatic failure, or renal failure are shown in Supplementary Table S1 (9).

Patients who passed the preliminary screening underwent a daily Spontaneous Awakening Trial (SAT). During this process, the analgesic and sedative infusions were interrupted until the patient awakened. Analgesics were continued for patients with active pain. Patients passed the SAT if they could perform all of the following three simple tasks: open their eyes, squeeze the examiner’s hand and move their fingers, and express discomfort. Patients failed the SAT if they developed persistent agitation, marked dyspnea, SPO_2_ < 88% for ≥5 min, or arrhythmias. Prior to randomization, clinicians administered sedatives and analgesics based on the patients’ conditions and the hospital’s prevailing practices. Clinicians titrated these medications to achieve the target analgesia level of −2–0 on the Critical-care Pain Observation Tool (CPOT), with specific drug choices being at the discretion of the hospital’s current resources without study intervention. Patients who were expected to be weaned after 12 h underwent a Spontaneous Breathing Trial (SBT) safety screening, and those who passed it were considered for inclusion in the final randomization study. A patient was considered to have passed the SBT safety screen if they demonstrated resolution or partial resolution of the underlying cause of respiratory failure; the ability to breathe spontaneously; adequate oxygenation (oxygen partial pressure ≥60 mmHg, fraction of inspired oxygen ≤40%, and positive end-expiratory pressure ≤8 cmH_2_O); a stable cardiovascular status without signs of myocardial ischemia or hypotension; and no or minimal requirement for vasopressors (dopamine or dobutamine ≤5 μg/kg/min or norepinephrine ≤0.05 μg/kg/min) (10).

### Randomization

Randomization was carried out 12 h prior to the anticipated extubation time. The random sequence was generated by the De Pai EDC (Electronic Data Collection) system, with eligible patients being randomly assigned in a 1:1 ratio to either group M or group R.

### Intervention

In group M, the sedative regimen was transitioned to midazolam, administered at a maintenance dose of 0.04 to 0.20 mg/kg/h. Conversely, group R patients were switched to remimazolam besylate, which was provided at a continuous maintenance infusion rate of 0.1 to 0.3 mg/kg/h. Patients who showed obvious restlessness during sedation were eligible for a supplementary bolus dose of midazolam (0.04 to 0.30 mg/kg) in group M or remimazolam besylate (0.1 mg/kg) in group R to achieve the desired level of sedation quickly. If the maximum dosage of the study drug was insufficient for adequate sedation (midazolam >0.20 mg/kg/h, remimazolam besylate>0.30 mg/kg/h), clinicians had the discretion to administer additional dexmedetomidine or propofol for rescue sedation. Sedative dosages were titrated by bedside nurses or physicians to maintain the target sedation level (Richmond Agitation-Sedation Scale [RASS] score of −3 to 0), with sedation depth being assessed every 4 h (or more frequently if necessary) using the RASS score. Any discrepancies were addressed through consultation with a third medical professional.

After enrollment, all patients continued to be managed with SAT and SBT protocols, as determined by the physician, approximately 12 h after the initiation of sequential sedation. On successful completion of the SAT, the patients were immediately subjected to a 30 min SBT trial with a pressure support of 5–8 cmH_2_O, positive end expiratory pressure (PEEP) of 5 cmH_2_O, and a fraction of inspired oxygen (FiO_2_) of 40%. An SBT trial was deemed unsuccessful if patients exhibited any of the following signs: respiratory rate > 35 breaths/min or < 8 breaths/min, hypoxemia (SPO_2_ or SaO_2_ < 90%), abrupt changes in mental status, unstable cardiovascular status with heart rate and blood pressure fluctuations exceeding 20% from baseline, acute cardiac arrhythmia, tachycardia (heart rate > 140 beats/min) or bradycardia (heart rate < 60 beats/min), shortness of breath, or evidence of increased respiratory effort, such as the use of accessory muscles or abdominal paradox.

### Outcomes

Upon successful completion of SBT, physicians decided to extubate the patients. The primary endpoint of this study was time to extubation, defined as the interval between the cessation of sedative administration and the removal of the endotracheal tube. The secondary endpoints included recovery time, defined as the duration from sedation cessation to the patient’s full awakening. Additionally, the incidence of agitation during the 4 h period following post-sedation cessation, the occurrence and duration of delirium, the length of ICUs and hospital stay, the proportion of time within the target RASS range, ICUs and hospital mortality, and adverse events were meticulously monitored and documented.

### Statistical analysis

Weaning time is the primary outcome. Based on previous studies (11), we assumed that weaning time would be reduced by 8 h in the group propofol compared with the group midazolam. Some studies have shown that remimazolam has comparable sedative effects to propofol (12). Calculating the standard deviation (22 h) by combining the variance of midazolam and propofol, a sample size of 244 patients from two groups was thus estimated to provide 80% power at a two-sided significance level of 0.05. Some patients may have withdrawn from the treatment, and 306 patients were enrolled, resulting in a 20% dropout rate.

SPSS 24.0 (IBM SPSS Statistics, Armonk, NY) was used for statistical analysis. Continuous variables were expressed as means ± standard deviations or medians with interquartile range (IQR), and categorical variables were expressed as numbers and percentages. The difference between groups M and R was compared using the Student’s *t*-test and the chi-squared test. The length of ICU stay was calculated using the log-rank test, and survival probabilities were depicted using the Kaplan–Meier method. All reported *p*-values were two-tailed, and a *p*-value of 0.05 was considered statistically significant.

Subgroup analyses were performed as post-hoc analyses. The difference in recovery time and extubation time between groups M and R was separately compared with subgroups of RASS, liver function, and age group. Finally, the linear regression model was applied to explore the risk factors for extubation time and recovery time, separately.

## Results

### Participants and baseline characteristics

**Figure 2:**
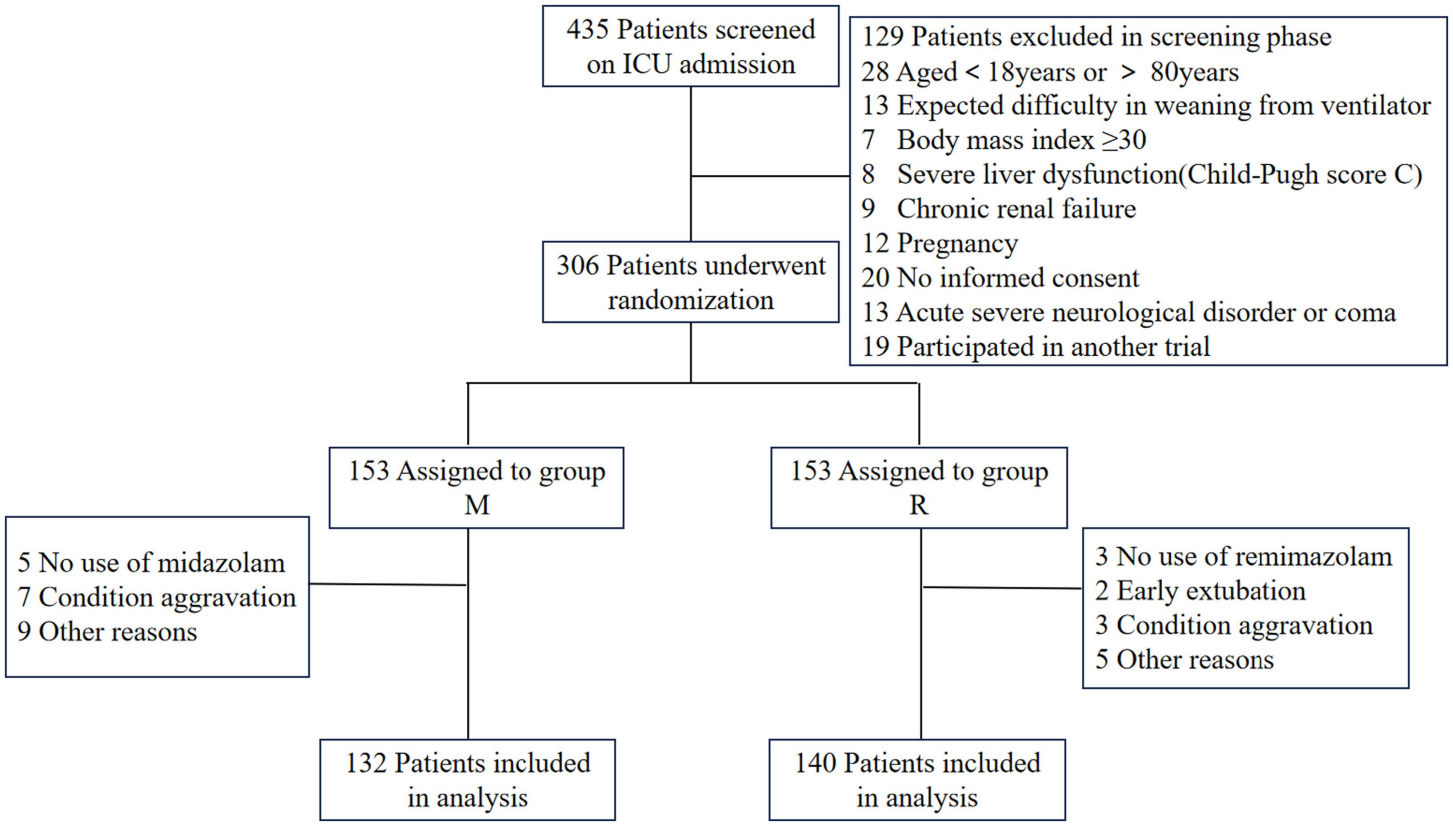
Patient screening, enrollment, and randomization.

A total of 435 patients were screened, of whom 306 patients were randomized, and 272 were ultimately included in the study, with 132 allocated to group M and 140 to group R (Figure 2). There were no significant differences in the baseline characteristics between the two groups, with the exception that group R had a higher proportion of male patients and individuals with impaired liver function at the time of enrollment (Table 1).

**Table 1:** Baseline characteristics of patients.

Characteristic	Group M (*n* = 132)	Group R (*n* = 140)	*P*-value
Age, mean (SD), year	55.95 (15.65)	57.48 (13.99)	0.397
Male, No. (%)	73 (55.30)	95 (67.86)	0.033*
BMI, mean (SD), kg/m^2^	23.65 (4.24)	23.58 (3.52)	0.875
Weight, mean (SD), kg	65.25 (13.75)	66.58 (12.45)	0.404
Apache II, median (IQR)	10 (7–16)	10 (6–18)	0.366
PaO_2_/FiO_2_, median (IQR), mmHg	270 (205–415)	280 (197–410)	0.358
Hepatic function at enrollment			
ALT, median (IQR), IU/L	23 (15–35)	23 (16–57)	0.048*
AST, median (IQR), IU/L	27 (20–45)	30 (21–55)	0.068
TBIL, median (IQR), umol/L	16.15 (11.1–24.6)	18.3 (12.1–27.2)	0.446
The ratio of impaired liver function at enrollment	30	42	0.032*
RASS score at enrollment, median (IQR)	−2 (−2–1)	−2 (−2–0)	0.019*
Diagnosis, No. (%)			0.148
Pneumonia	9	9	
Pancreatitis	5	2	
Trauma	33	54	
Sepsis	3	3	
Other diseases	82	72	
Use of vasopressors, No. (%)			0.400
Yes	38	34	
No	94	106	
Previous medical history, No. (%)			0.686
Yes	73	74	
No	59	66	
Smoke			0.587
Yes	26	24	
No	106	116	

*: *p* < 0.05; **: *p* < 0.01; ***: *p* < 0.000; APACHE II, acute physiology and chronic health evaluation II; ALT, alanine aminotransferase; AST, aspartate aminotransferase; TBil, total bilirubin.

### Study outcomes

**Figure 3:**
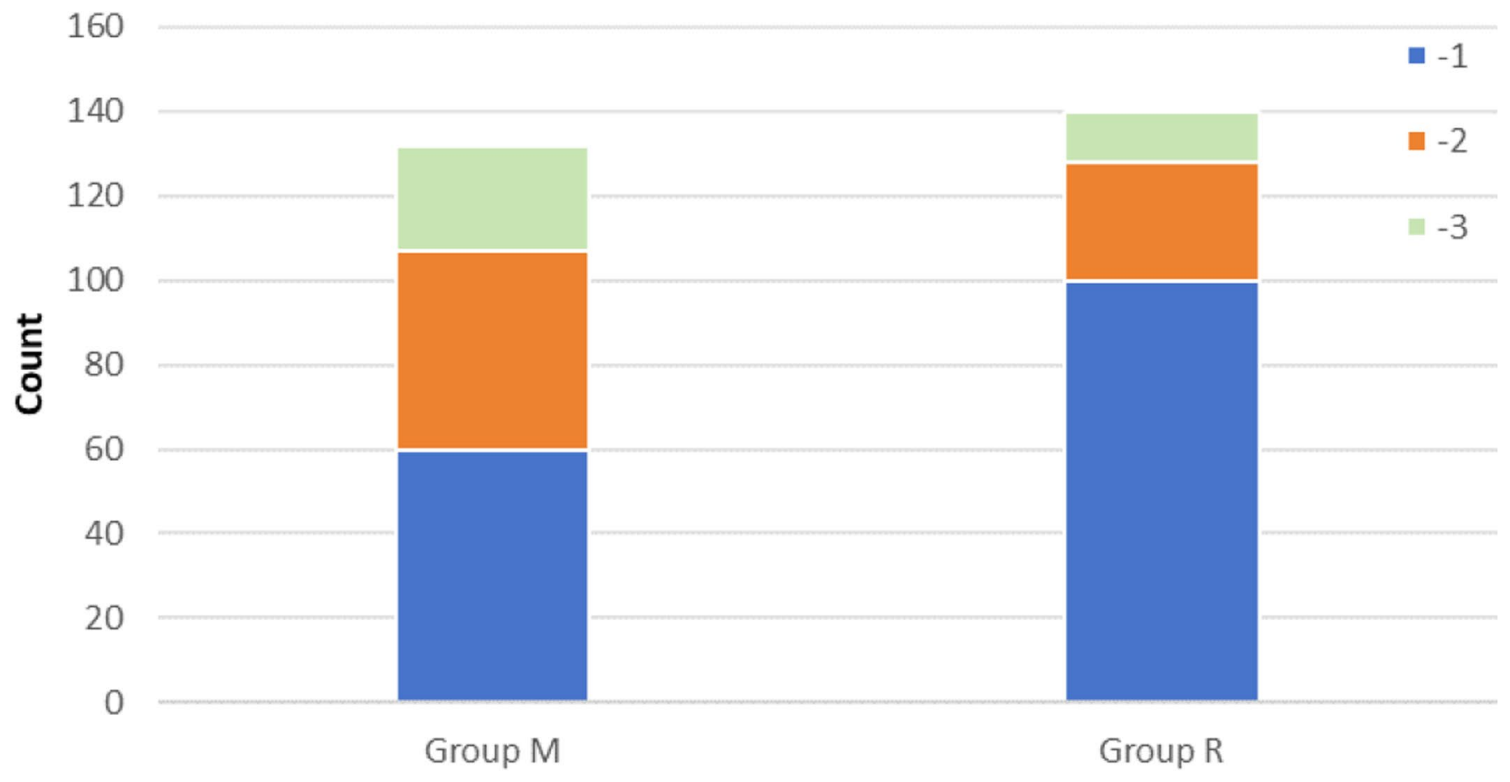
Number of patients with RASS score before stopping sedatives between groups M and R.

In the primary outcome analysis, both recovery time and extubation time were significantly shorter in group R compared to group M (both *p* < 0.001, Table 2). Group R maintained a lighter level of sedation than group M (Table 2; Figure 3). Furthermore, patients in group R demonstrated rapid recovery (*p* < 0.05) and extubation (*p* < 0.05), with the same RASS score before the cessation of sedatives (Tables 3, 4; Supplementary Figure S1). Subgroup analyses found that abnormal liver function led to longer recovery time and extubation times. However, within these subgroups, the time in group R was shorter than that in group M (Table 5). Similarly, in older patients (age >70 years), group M had a longer recovery time compared to group R (Supplementary Table S2). Further exploration of risk factors for extubation and recovery time using a linear regression model showed that increased recovery and extubation time were correlated with group M, higher APACHE-II scores (Supplementary Tables S3–S6). There were no significant differences between the groups in successful extubation, no mechanical ventilation within 28 days, or mortality during the ICU and hospital stay (Table 2; Supplementary Figure S2).

**Table 2:** Study outcomes.

Outcome	Group M (*n* = 132)	Group R (*n* = 140)	*P*-value
Adjust the sedation plan, No. (%)			0.582
Yes	4	6	
No	128	134	
Extubation for first time, No. (%)			
Success	130	139	0.527
Failure	2	1	
Recovery time median (IQR), min	20 (10–45)	5 (4–10)	0.000***
Extubation time, median (IQR), min	80 (55–120)	51 (41–80)	0.000***
Percentage of times within target sedation range, %	100 (95–100)	90 (90–100)	0.000***
ICU duration, median (IQR), day	3 (2–6)	3 (2–5)	0.078
Length of hospital stay, median (IQR), day	12 (8–20)	14 (9–20)	0.146
None MV within 28 days	27 (24–27)	27 (26–27)	0.415
ICU mortality, No. (%)	5 (3.79)	1 (0.71)	0.085
Delirium, No. (%)	6 (4.55)	4 (2.86)	0.460
Agitation, No. (%)	27 (20.45)	12 (8.57)	0.005**
Duration of MV, median (IQR), day	1 (0.9–3)	1 (0.9–2)	0.241
The maintenance of sedation, median (IQR), mg/kg/h	0.09 (0.07–0.1)	0.16 (0.1–0.2)	0.000***
RASS in sedation	−2 (−2–−1)	−1 (−2–−1)	0.000***
Hepatic function After extubation			
ALT, median (IQR), IU/L	23 (15–42)	24 (16–48)	0.417
AST, median (IQR), IU/L	28 (19–53)	26 (19–51)	0.836
TBIL, median (IQR), umol/l	15 (10–23)	15 (10–24)	0.109
The ratio of impaired liver function	34 (25.76)	37 (26.43)	0.900
Sedative duration before sequential sedation (h)	8 (5.25–22.75)	8.25 (5.25–24)	0.144

*: *p* < 0.05; **: *p* < 0.01; ***: *p* < 0.000.

**Table 3:** Comparison of recovery time at different sedation levels before stopping sedatives.

RASS	Group M	Group R	*P*-value
−1	10 (6–16)	5 (3–7)	0.016
−2	25 (18–45)	11 (9–16)	0.000
−3	80 (55–180)	25 (18–30)	0.012

Recovery time median (IQR), min.

**Table 4:** Comparison of extubation time at different sedation levels before stopping sedatives.

RASS	Group M	Group R	*P-*value
−1	62 (42–90)	50 (40–71)	0.049
−2	76 (60–120)	50 (45–93)	0.027
−3	130 (106–367)	62 (53–105)	0.004

Extubation time median (IQR), min.

**Table 5:** Effect of liver function on recovery time and extubation time.

Outcome	Normal liver function			Hepatic dysfunction		
	Group M	Group R	*P*-value	Group M	Group R	*P*-value
Recovery time	19 (10–39)	5 (4–12)	0.003	29 (10–60)	6 (3–10)	0.040
Extubation time	79 (55–120)	50 (41–79)	0.000	85 (50–127)	57 (40–85)	0.039

### Sedation efficacy and adverse events

No significant difference was observed in the median sedation duration before the sequential sedation protocol between group M (8 h, interquartile range [IQR], 5.25 to 22.75 h) and group R (8.25 h, IQR, 5.25 to 24 h) (*p* = 0.144). After randomization, group M demonstrated a higher percentage of time within the targeted sedation range compared to group R (100% [95, 100%] versus 90% (90%, 100%), *p* < 0.001; Table 2). The infusion dosages of the study drugs are presented in Table 2, and no significant difference was found in the requirement for rescue sedation. A higher incidence of agitation was observed in group M compared to group R (20.45% versus 8.57%, *p* = 0.005), while the incidence of delirium did not show a significant difference between the two groups (Table 2). There were no significant adverse events that occurred.

## Discussion

Our study showed that switching to remimazolam besylate prior to sedative withdrawal was associated with faster recovery, earlier extubation, and a less frequent incidence of agitation compared to midazolam. To the best of our knowledge, this is the first study to evaluate the sequential use of midazolam and remimazolam besylate in combination with the mechanical ventilation weaning process in critically ill patients. Despite the emergence of many new sedatives (5, 13), midazolam remains one of the most frequently prescribed medications, being used in up to 62% of elderly patients requiring mechanical ventilation for sedation (14). Guidelines currently recommend targeting light sedation in adults receiving mechanical ventilation (2). However, applying light sedation with benzodiazepine medications to medical practice and achieving optimum sedation in long-term sedation remains a clinical challenge due to the risk of drug accumulation.

Remimazolam besylate is a novel, ultra-short-acting benzodiazepine that is rapidly metabolized by non-specific tissue esterases into an inactive carboxylic acid metabolite, thereby presenting a rapid and a predictable onset and offset profile (15). Meanwhile, previous trials have reported that the use of short-acting sedatives before weaning from mechanical ventilation is associated with faster recovery and early extubation, as well as lower ICU treatment costs. In this study, a median remimazolam besylate infusion rate of 0.18 mg/kg/h provided light-to-moderate sedation (16), which is a safe and effective sedative for procedural sedation due to its higher procedural success rate, faster recovery, shorter discharge time, and superior safety profile compared to traditional sedatives (17). In accordance with previous studies (16), the goal of sedation target level was a RASS score of 0 to −3 after randomization. The median remimazolam besylate infusion rate in this study was 0.16 mg/kg/h, and the proportion of time at target sedation level was higher in group M than in group R. This study found that remimazolam, when used during the period of mechanical ventilation, provided light sedation more readily than midazolam. Patients who were sequentially treated with remimazolam showed faster recovery and early extubation. We further compared the differences in recovery and extubation times between the two groups at the same level of RASS and arrived at the same conclusion. Considering the variations in sedation duration before randomization that might influence outcomes, we compared the sedation duration before randomization and found no statistical difference between the groups. Various physiological parameters, including age-related effects, compromised renal function, and liver dysfunction, may affect the pharmacokinetics of benzodiazepine medications (18). In this study, abnormal liver function and advanced age appeared to have minimal impact on the recovery and extubation times in group R compared to group M, and renal insufficiency was not analyzed due to the limited number of cases.

ICU patients with long-term exposure to benzodiazepine sedatives may experience withdrawal symptoms (19). Midazolam, a water-soluble benzodiazepine with a rapid onset and short duration of action, can cause withdrawal syndrome, including agitation, immediately following cessation (20). In our study, patients in the midazolam group had a higher incidence of agitation than those in the remimazolam group. Clinically, this agitation seemed to be related to deeper sedation, with patients taking longer to recover from deeper sedation and being more prone to agitation during this transition. There was no statistically significant difference in the incidence of delirium between the two groups.

As the conception of light sedation has been widely accepted in recent years, sedatives have been titrated to achieve the goal of light sedation in our usual care whenever possible. This study demonstrates that remimazolam, as a new benzodiazepine, retains both its advantages and none of the adverse reactions of other sedative drugs and may become a new strategy for sedation of mechanically ventilated patients in the ICUs, which needs to be further confirmed by larger clinical studies.

This study has several limitations. First, as a multicenter trial, we cannot use uniform analgesic types during sequential sedation according to the concrete condition in China, as each medical center has a variety of analgesics available. Second, the use of different sedative drugs before randomization might influence outcomes. Third, the majority of participants in our study were postoperative patients, and the duration of mechanical ventilation was comparatively shorter than that reported in other investigations. Additionally, we only screened mechanically ventilated patients who should be weaned from mechanical ventilation, and only patients with complete follow-up results were analyzed.

## Conclusion

In critically ill, mechanically ventilated patients, the use of remimazolam besylate was associated with a shorter time to recovery, early extubation, and a lower incidence of agitation. These findings indicate that the sequential use of remimazolam besylate was a novel sedation strategy that might provide clinically relevant benefits for selected critically ill, mechanically ventilated patients.

## Data Availability

The raw data supporting the conclusions of this article will be made available by the authors, without undue reservation.
