# Correction: Effect of remimazolam besylate versus midazolam on time to extubation in critically ill, mechanically ventilated patients: a randomized controlled trial

**DOI:** 10.3389/fmed.2025.1695118

**Published:** 2025-09-02

**Authors:** Frontiers Production Office

**Affiliations:** Frontiers Media SA, Lausanne, Switzerland

**Keywords:** remimazolam besylate, midazolam, sequential sedation, intensive care, mechanical ventilation

The incorrect citation was cited in **Discussion**, paragraph two. It should be reference 16 instead of 18. The corrected sentence appears below:

“In accordance with previous studies (16), the goal of sedation target level was a RASS score of 0 to −3 after randomization.”

The incorrect citation was cited in **Discussion**, paragraph two. It should be reference 18 instead of 16. The corrected sentence appears below:

“Considering the variations in sedation duration before randomization that might influence outcomes, we compared the sedation duration before randomization and found no statistical difference between the groups. Various physiological parameters, including age-related effects, compromised renal function, and liver dysfunction, may affect the pharmacokinetics of benzodiazepine medications (18).”

The reference details for references 18 and 20 should be swapped. The correct references Spina et al. (18) and Sanavia et al. (20) appear below:

18. Spina, SP, Ensom, MH. Clinical pharmacokinetic monitoring of midazolam in critically ill patients. *Pharmacotherapy*. (2007) 27:389–98. doi: 10.1592/phco.27.3.389

20. Sanavia, E, Mencía, S, Lafever, SN, Solana, MJ, Garcia, M, and López-Herce, J. Sedative and analgesic drug rotation protocol in critically ill children with prolonged sedation: evaluation of implementation and efficacy to reduce withdrawal syndrome. *Pediatr Crit Care Med*. (2019) 20:1111–7. doi: 10.1097/PCC.0000000000002071

The original version of this article has been updated.

