# Supplementary material for: Effect of remimazolam besylate versus midazolam on time to extubation in critically ill, mechanically ventilated patients: a randomized controlled trial
Source: Front Med (Lausanne). 2025 Aug 26;12:1553495. doi: 10.3389/fmed.2025.1553495 (PMC12439343; doi:10.3389/fmed.2025.1553495)

Table S1.

| Chronic hepatic failure: decompensated chronic liver disease with premorbid Child-Pugh class B or C. |
| --- |
| Chronic renal failure: persistent renal impairment with serum creatinine elevation for more than three months or a calculated glomerular filtration rate (GFR) below 60 ml/min/1.73m^2^. |
| Liver injury criterion   - ALT≥5×ULN - ALP≥2×ULN without findings of skeletal disease(elevated serum ALP) - ALT≥3×ULN and TBil≥2×ULN |
| AlP, alkaline phosphatase; ALT, alanine aminotransferase; AST, aspartate  aminotransferase; ULN, upper limit of normal; TBil, Total bilirum. |

Table S2. The effect of age on recovery time and extubation time

| Outcome | age≤70 years | | |  | age>70 years | | |
| --- | --- | --- | --- | --- | --- | --- | --- |
|  | Group M | Group R | *P* value |  | Group M | Group R | *P* value |
| Recovery time | 19(10-48) | 5(3-10) | 0.001 |  | 28(13-37) | 7(5-16) | 0.022 |
| Extubation time | 81(55-120) | 51(40-80) | 0.000 |  | 74(60-120) | 56(45-78) | 0.029 |

Table S3. Univariate liner regression model to explore the risk factor for extubation time

|  | Coef. | *P* | 95%CI |
| --- | --- | --- | --- |
| Group M/R | -60.37 | 0.000 | -88.34-32.40 |
| Age | 0.39 | 0.429 | -0.58- -1.37 |
| Gender(Male/Female) | -11.83 | 0.433 | -41.51- -17.85 |
| BMI | -2.80 | 0.140 | -6.51- -0.92 |
| Liver function(Abnormal/normal) | 6.26 | 0.708 | -26.61- -39.13 |
| APACHEII | 3.16 | 0.001 | 1.29-5.03 |

Table S4. Multivariable liner regression model to explore the risk factor for extubation time

|  | Coef. | *P* | 95%CI |
| --- | --- | --- | --- |
| Group M/R | -66.11 | 0.000 | -94.2- -38.11 |
| Age | 0.06 | 0.905 | -0.93-1.05 |
| Gender(Male/Female) | -18.45 | 0.215 | -47.68-10.79 |
| BMI | -2.50 | 0.174 | -6.11-1.11 |
| Liver function(Abnormal/normal) | -2.36 | 0.884 | -34.36-29.63 |
| APACHEII | 3.23 | 0.001 | 1.32-5.15 |

Table S5. Univariate liner regression model to explore the risk factor for recovery time

|  | Coef. | *P* | 95%CI |
| --- | --- | --- | --- |
| Group M/R | -47.13 | 0.000 | -72.19- -22.07 |
| Age | 0.28 | 0.533 | -0.59-1.14 |
| Gender(Male/Female) | -11.79 | 0.380 | -38.17-14.59 |
| BMI | -2.27 | 0.177 | -5.57-1.03 |
| Liver function(Abnormal/normal) | 7.27 | 0.625 | -21.95-36.49 |
| APACHEII | 2.29 | 0.008 | 0.62-3.97 |

Table S6. Multivariable liner regression model to explore the risk factor for recovery time

|  | Coef. | *P* | 95%CI |
| --- | --- | --- | --- |
| Group M/R | -52.11 | 0.000 | -77.42- -26.8 |
| Age | 0.06 | 0.892 | -0.84-0.96 |
| Gender(Male/Female) | -17.32 | 0.198 | -43.74-9.09 |
| BMI | -2.04 | 0.219 | -5.30-1.22 |
| Liver function(Abnormal/normal) | 0.84 | 0.954 | -28.07-29.76 |
| APACHEII | 2.31 | 0.009 | 0.58-4.04 |

Figure S1.Extubation time in group M and group R.

Figure S2. Kaplan-Meier plot of length of stay in the intensive care unit and number of patients at risk from start of study drug to 28 days (Log-rank P=0.375).


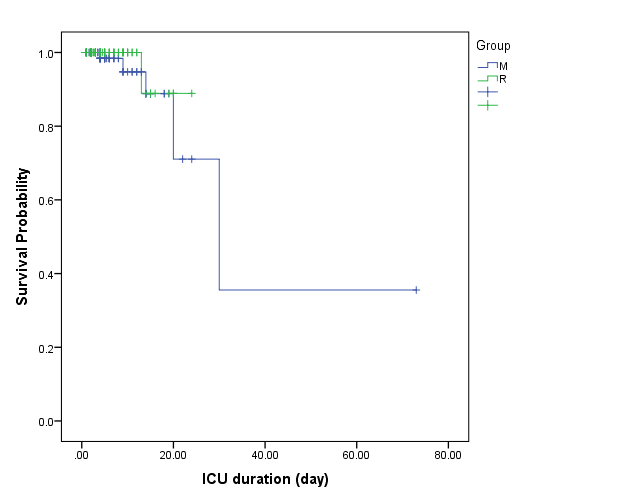

Supplement: Supplementary file 1 [file Data_Sheet_1.docx]
